# C‐type natriuretic peptide induces inotropic and lusitropic effects in human 3D‐engineered cardiac tissue: Implications for the regulation of cardiac function in humans

**DOI:** 10.1113/EP091303

**Published:** 2023-07-26

**Authors:** Julian C. Bachmann, Jeppe E. Kirchhoff, Julia E. Napolitano, Steve Sorota, William M. Gordon, Nicole Feric, Roozbeh Aschar‐Sobbi, Juan Lv, Zhiyou Cao, Ken Coppieters, Giulia Borghetti, Michael Nyberg

**Affiliations:** ^1^ Research & Early Development Novo Nordisk A/S Maaloev Denmark; ^2^ Valo Health Boston MA USA

**Keywords:** contractility, human stem cell‐derived cardiomyocytes, Langendorff isolated heart, relaxation, translation

## Abstract

The role of C‐type natriuretic peptide (CNP) in the regulation of cardiac function in humans remains to be established as previous investigations have been confined to animal model systems. Here, we used well‐characterized engineered cardiac tissues (ECTs) generated from human stem cell‐derived cardiomyocytes and fibroblasts to study the acute effects of CNP on contractility. Application of CNP elicited a positive inotropic response as evidenced by increases in maximum twitch amplitude, maximum contraction slope and maximum calcium amplitude. This inotropic response was accompanied by a positive lusitropic response as demonstrated by reductions in time from peak contraction to 90% of relaxation and time from peak calcium transient to 90% of decay that paralleled increases in maximum contraction decay slope and maximum calcium decay slope. To establish translatability, CNP‐induced changes in contractility were also assessed in rat *ex vivo* (isolated heart) and in vivo models. Here, the effects on force kinetics observed in ECTs mirrored those observed in both the *ex vivo* and in vivo model systems, whereas the increase in maximal force generation with CNP application was only detected in ECTs. In conclusion, CNP induces a positive inotropic and lusitropic response in ECTs, thus supporting an important role for CNP in the regulation of human cardiac function. The high degree of translatability between ECTs, *ex vivo* and in vivo models further supports a regulatory role for CNP and expands the current understanding of the translational value of human ECTs.

## Abstract (highlights)

- **What is the central question of this study?**
  What are the acute responses to C‐type natriuretic peptide (CNP) in human‐engineered cardiac tissues (ECTs) on cardiac function and how well do they translate to matched concentrations in animal *ex vivo* and in vivo models?
- **What is the main finding and its importance?**
  Acute stimulation of ECTs with CNP induced positive lusitropic and inotropic effects on cardiac contractility, which closely reflected the changes observed in rat *ex vivo* and in vivo cardiac models. These findings support an important role for CNP in the regulation of human cardiac function and highlight the translational value of ECTs.

## 1 INTRODUCTION

C‐type natriuretic peptide (CNP) is released by endothelial cells, fibroblasts and cardiomyocytes in the heart where it elicits autocrine and paracrine signalling via its interaction with natriuretic peptide receptor (NPR) 2 and NPR3 (Cerra & Pellegrino, 2007; Moyes & Hobbs, 2019; Nyberg et al., 2023). NPR2 is a particulate guanylyl cyclase that catalyses the synthesis of cyclic guanosine monophosphate (cGMP) whereas NPR3 clears CNP via a receptor‐mediated internalization and degradation process, but a signalling function (G_i_ protein‐linked receptor) has also been reported (Moyes & Hobbs, 2019). Following ligation of NPR2, the resulting increase in intracellular cGMP and subsequent increase in protein kinase G (PKG) activity leads to phosphorylation of phospholamban (regulator of sarco/endoplasmic reticulum Ca^2+^‐ATPase 2 (SERCA2) controlling the influx of calcium to the SR), cardiac troponin I (regulatory protein that controls calcium‐mediated interaction between actin and myosin) and titin (controlling cardiomyocyte stiffness) (Moyes & Hobbs, 2019; Sangaralingham et al., 2023).

Findings in murine in vitro, *ex vivo* and in vivo systems have revealed a direct positive lusitropic effect of CNP (Beaulieu et al., 1997; Manfra et al., 2022; Moltzau et al., 2013, 2014; Qvigstad et al., 2010), whereas some controversy exist regarding its effect on force generation as both increases (Beaulieu et al., 1997; Hirose et al., 1998) and decreases (Manfra et al., 2022; Moltzau et al., 2013, 2014; Qvigstad et al., 2010) in inotropy have been reported. Importantly, due to distinct species differences in cardiac structure and function between rodents and humans (Kusunose et al., 2012; Milani‐Nejad & Janssen, 2014; Popovic et al., 2005), caution should be taken when translating mechanistic findings in rodent models into humans. Hence, since insight into the role of CNP for the regulation of cardiac function has been confined to observations from animal models, mechanistic studies in human systems are warranted to provide clarity on the human relevance of these findings.

Human 3D cardiac microphysiological systems, including engineered cardiac tissues (ECTs) using human induced pluripotent stem cell (hiPSC)‐derived cardiomyocytes, are gaining significant interest given the increasing need for complex in vitro model systems to improve translatability and consequently also replace animal testing. By utilizing the Biowire II Platform, 3D ECTs can be generated from hiPSC‐derived cardiomyocytes and cardiac fibroblasts. These ECTs display a phenotype that mimics adult human myocardium including a lack of spontaneous beating, the presence of a positive force–frequency response from 1 to 4 Hz and prominent post‐rest potentiation. Furthermore, canonical responses to pharmacological agents that affect contractility in humans via a variety of mechanisms are also evident in this model system (Feric et al., 2019). Accordingly, this model is suitable for assessing human‐relevant effects of CNP on contractility.

In the present study, we leveraged ECTs to study the acute effects of CNP on contractility in a well‐characterized human 3D system. To provide insight into the translatability between animal findings and those obtained in the present in vitro model system, CNP‐induced changes in contractility were assessed in rat *ex vivo* and in vivo models in which circulating concentrations of CNP were matched to those applied in ECTs.

## 2 METHODS

### 2.1 Ethics approval

The experiments were conducted in part in Denmark under a license from the Danish Ministry of Justice (License No. 2020‐15‐0201‐00529 and 2018‐12‐MJXU) and in part in China under licenses granted by the Beijing Administration Office of Laboratory Animals (BAOLA) (License No. SYXK(京)2018‐0017). All animal experiments were approved by the internal Novo Nordisk Ethical Review Committee in Denmark and conducted in accordance with the practical guidelines for rigor and reproducibility in preclinical studies on cardio‐protection (Botker et al., 2018).

### 2.2 Synthesis of CNP

CNP22 was synthesized by solid‐phase peptide synthesis using standard fluorenylmethoxycarbonyl (Fmoc)‐based amino acids and reagents. Amino acid couplings were performed using four equivalents of Fmoc‐protected amino acid, *N*,*N*′‐diisopropylcarbodiimide (DIC) and Oxyma Pure, respectively, in dimethylformamide (DMF) for 90 min. After coupling, the resin was capped by treatment with 1.2 M acetic anhydride and 0.6 M *N*,*N*‐diisopropylethylamine (DIPEA) in DMF for 30 min. Fmoc groups were removed by treatment with 20% piperidine in DMF for 2 × 15 min. The peptide was cleaved from the resin by treatment with trifluoroacetic acid, triisopropylsilane, 1,4‐dithiothreitol and water (90:5:2.5:2.5) in addition to ammonium iodide at 15 mg/ml cleavage mixture. The crude peptide was purified by Reversed‐phase high‐performance liquid chromatography (RP‐HPLC) using a gradient of acetonitrile and water with 0.1% trifluoroacetid acid. The peptide was cyclized by the addition of 1.0 equivalent of 4‐aldrithiol. The cyclized peptide was purified using RP‐HPLC as described above and lyophilized to yield the peptide as a colourless powder.

### 2.3 Study protocol: in vitro experiments

**FIGURE 1:**
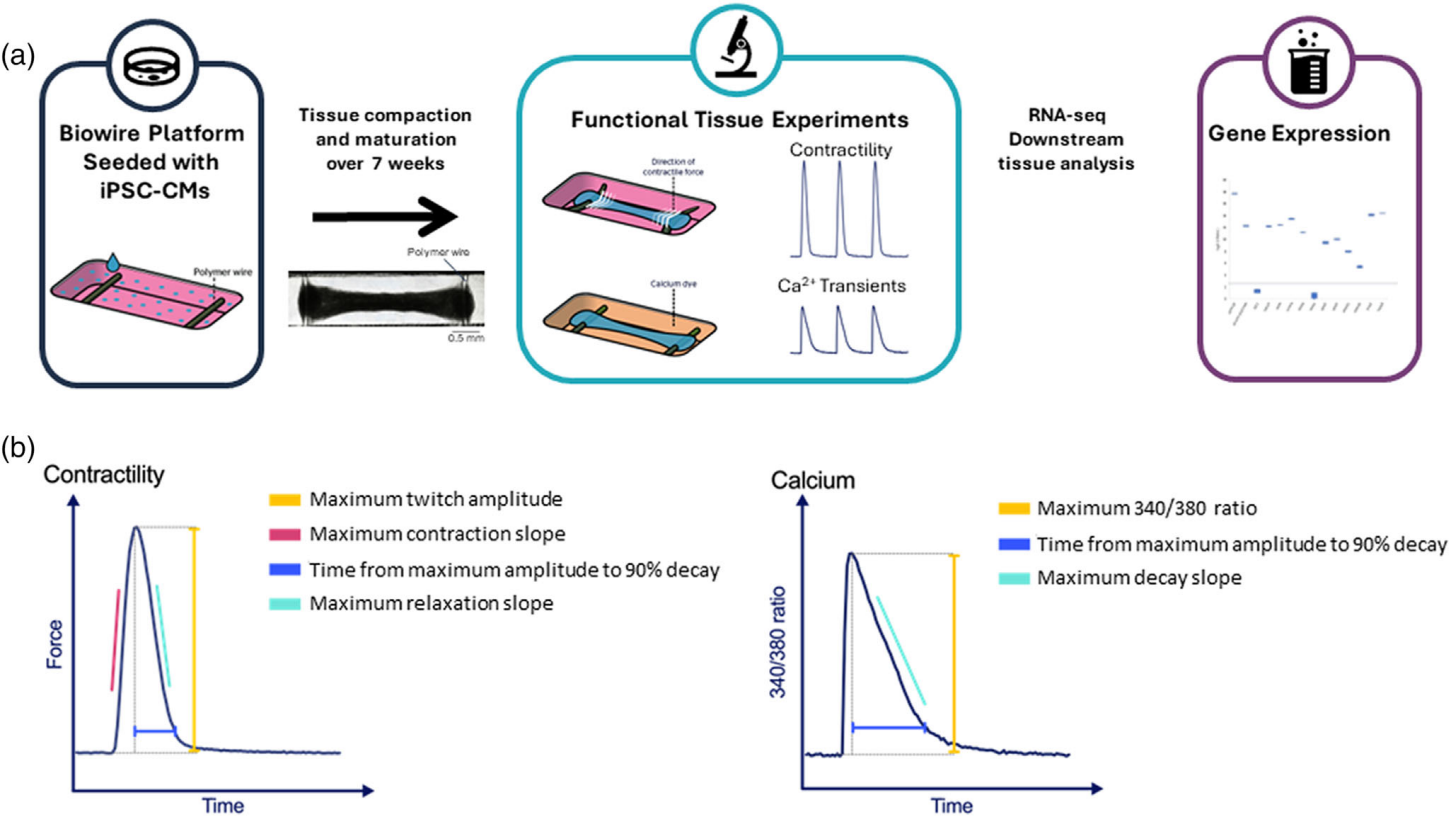
Illustration of the engineered cardiac tissue model and measured variables. (a) A representative image of an ECT in the Biowire platform attached to the polymer wires. Twitch force is measured by recording a video of the contracting tissues under field stimulation and converting pixel displacement of the polymer wires into force measurements. (b) Measured parameters for contractility and calcium transients. CM, cardiomyocyte; ECT, engineered cardiac tissue.

3D ECTs were generated with the Biowire platform as previously described (Zhao et al., 2019). In short, ECTs were formed in polystyrene microwells containing parallel poly (octamethylene maleate (anhydride) citrate) (POMaC) wires. For each tissue, 100,000 iPSC‐derived cardiomyocytes (iCell® Cardiomyocytes^2^, Fujifilm Cellular Dynamics International, Madison, WI, USA) and 10,000 human ventricular cardiac fibroblasts (Lonza, Allendale, NJ, USA) in a collagen/Matrigel/fibrin gel were seeded in each well. After 7 days of culture, the cardiomyocytes and fibroblasts formed 3D ECTs around the polymer wires (Figure 1). Custom chambers containing parallel carbon electrodes were used to provide electrical field stimulation using biphasic pulses of 2 ms duration, at twice the excitation threshold. Stimulation was started at 1 Hz and increased by 0.1 Hz increments daily to a maximum of 6 Hz. The ECTs used in this study were matured for 7 weeks in the Biowire II platform.

For calcium and contractility measurements, ECTs were stained with 5 μM Fura‐4F‐AM (Thermo Fisher Scientific, Waltham, MA, USA) for 45 min in modified Krebs–Henseleit buffer (Millipore Sigma (Burlington, MA, USA) cat. no. K3753, containing 118 mM NaCl, 4.7 mM KCl, 1.2 mM Mg_2_SO_4_, 1.2 mM KH_2_PO_4_, 11 mM glucose, 22 mM NaHCO_3_ and 1.8 mM CaCl_2_) at 37°C and 5% CO_2_ (pH 7.4). Tissues were then placed in fresh buffer and incubated for 15 min prior to baseline recording. Calcium transients were recorded at alternating 340/380 nm excitation, and emission was measured at 510 nm using Andor Xyla 4.2 (Andor, South Windsor, CT, USA) camera at 100 frames per second using NIS‐Elements Advanced Research software (Nikon Instruments Inc., Edgewood, NY, USA). Contractility was measured by tracking the deflection of POMaC wires as previously described (Zhao et al., 2019). Briefly, the polymer wires were illuminated using 480 nm excitation, and videos were recorded at 100 frames per second at 515 nm emission. The pixel displacement of the polymer wires was converted to force using custom software to determine the amplitude and kinetics of contractility (Figure 1). CNP was added to the recording chamber at increasing doses (10 and 100 nM) with 15 min incubation per dose, after which contractility and calcium measurements were acquired from each tissue. At the end of each experiment, 100 nM isoproterenol (ISO, Cayman Chemical, Ann Arbor, MI, USA) was added to each tissue as a positive control for tissue responsiveness, and only tissues displaying a canonical response were included in the data.

The isolated effects of ISO and dobutamine at three doses were also tested in separate experiments, as cardiac dynamics following the application of these compounds are well described in humans and preclinical models.

### 2.4 Study protocol: *ex vivo* experiments

A total of 29 male Sprague–Dawley rats (Charles River, Romans, France) at 10–12 weeks of age were housed three to four per each cage in a temperature‐ and humidity‐controlled environment under a 12/12‐h light–dark cycle (lights off 18.00 h) with ad libitum access to food (Altromin 1324, Altromin, Lage, Germany) and tap water. Rats then acclimated to their new environment for at least 1 week prior to testing.

Animals were anaesthetized by inhalation of 3% isoflurane in 0.6 litres/min O_2_. The aorta was cannulated directly in the animal and the heart was quickly placed in the Langendorff apparatus (Hugo Sachs‐Harvard Apparatus GmbH, March, Germany) and perfused retrogradely in a constant flow mode during stabilization in Krebs–Henseleit buffer with the following concentrations: 120.4 mM NaCl, 4.0 mM KCl, 2.5 mM CaCl_2_, 0.6 mM MgSO_4_, 15.3 mM NaHCO_3_, 0.6 mM NaH_2_PO_4_ and 11.5 mM glucose, pH 7.4, at constant oxygenation with O_2_/CO_2_ in a 95%–5% mixture at 37°C. The heart rate was kept constant at 300 bpm by right atrial pacing.

A water–alcohol‐filled latex balloon was inserted into left ventricle via the mitral valve. The distal end of the balloon catheter (Harvard Apparatus GmbH) was connected to a pressure transducer for measurement of left ventricular pressure (LVP), and all data recordings were acquired using LabChart (ADInstruments Ltd, United Kingdoms, Version 8.1.19). The balloon was inflated to a diastolic pressure of 10 mmHg. The experiments were conducted at a constant aortic pressure of 80 mmHg with variable flow. Ischaemia–reperfusion (IR) injury was induced by turning off the perfusion pump for 17.5 min.

CNP was dissolved in a vehicle consisting of 50 mM phosphate, 70 mM sodium chloride and 0.007% (30 nmol/ml) polysorbate 20. During the experiments, CNP was dissolved in the Krebs–Henseleit buffer to a final concentration of 30 nM. CNP or vehicle was added after 20 min of baseline measurements to investigate effects both before and after IR.

### 2.5 Study protocol: in vivo experiments

Male Sprague–Dawley rats, 7–8 weeks old (*n* = 13; Vital River Laboratory Animal Technology Co. Ltd, Beijing, China), were randomly assigned to groups upon arrival. Animals were allowed an acclimatization period for at least 2 weeks before entering the study. The rats had unlimited access to tap water and standard chow and all animals were housed in an enriched environment with standard bedding and nesting material, under a 12/12‐h day–night cycle (lights on at 06.00 h) in a humidity (30−70%) and temperature (20−25°C)‐controlled facility.

The surgical procedures were performed under anaesthesia with isoflurane (4–5% for induction and 2% for maintenance) and only after complete absence of reflexes. Animals were placed on controlled heating pads, and body temperature, measured via a rectal probe, was maintained at 37°C. An incision was then made in the left inguinal area along the natural angle of the hind leg, after which blunt dissection of the tissue was performed to expose the femoral artery. A pressure catheter (Scisense 1.2F solid‐state pressure catheter, Transconic, Ithaca, NY, USA) was inserted into the left femoral artery using a 25–30‐G needle to allow for arterial measurement of blood pressure. Then, an incision over the right carotid artery from mandible to sternum was performed to expose the carotid artery. The vagus nerve was gently dissected away and sterile 6‐0 sutures were placed around the proximal and distal parts of the carotid artery after which a pressure catheter (Scisense 1.2F solid‐state pressure catheter, Transconic) was inserted using a 25–30‐G needle. The catheter was gently advanced into the left ventricle and then secured by the tightening of the sutures. After stabilization for 10–15 min, haemodynamic parameters were acquired (SP200 Pressure System, Transconic) and recorded (Biopac MP160, Biopac systems, Inc., Goleta, CA, USA) at baseline (−15 to 0 min) and during constant infusion of CNP (200 pmol/min/kg) via the tail vein for 30 min. The infusion rate of CNP was based on calculations (one compartment model) of the dose needed to obtain a concentration of ∼10 nmol/l at steady state.

### 2.6 Analytical procedures

#### 2.6.1 *Ex vivo* and in vivo

The maximal slope of the systolic pressure increment and the diastolic pressure decrement was calculated and presented as d*P*/d*t*_max_ and d*P*/d*t*_min_. To provide further insight into diastolic function, the left ventricular relaxation time constant, tau, was also calculated. Left ventricular developed pressure (LVDP) was obtained by subtracting left ventricular end‐systolic pressure (LVESP) with left ventricular end diastolic pressure (LVEDP), and rate pressure product (RPP) was calculated by multiplying heart rate (HR) by systolic blood pressure.

### 2.7 RNA sequencing

#### 2.7.1 Biowire sample prep for RNAseq

Each Biowire tissue was placed directly in 100 μl of Trizol (Thermo Fisher Scientific) and flash frozen. RNA extraction, library preparation, sequencing and analysis were conducted at Azenta Life Sciences (South Plainfield, NJ, USA) as follows.

#### 2.7.2 RNA extraction and quality control

Total RNA was extracted from fresh frozen tissue samples using the Qiagen RNeasy Plus Universal mini kit following manufacturer's instructions (Qiagen, Hilden, Germany). RNA samples were quantified using Qubit 2.0 fluorometer (Thermo Fisher Scientific) and RNA integrity was checked using Agilent TapeStation 4200 (Agilent Technologies, Santa Clara, CA, USA).

#### 2.7.3 Library preparation

RNA sequencing libraries were prepared using the NEBNext Ultra RNA Library Prep for Illumina using manufacturer's instructions (NEB, Ipswich, MA, USA). Briefly, mRNAs were initially enriched with Oligod(T) beads. Enriched mRNAs were fragmented for 15 min at 94°C. First‐ and second‐strand cDNA were subsequently synthesized. cDNA fragments were end‐repaired and adenylated at 3′ ends, and universal adapters were ligated to cDNA fragments, followed by index addition and library enrichment by PCR. Sequencing libraries were validated on the Agilent TapeStation, and quantified using Qubit 2.0 fluorometer and by quantitative PCR (KAPA Biosystems, Wilmington, MA, USA).

#### 2.7.4 Illumina sequencing

Sequencing libraries were clustered on a lane of a HiSeq flowcell, and after clustering the flowcell was loaded on the Illumina instrument (4000 or equivalent) according to the manufacturer's instructions. The samples were sequenced using a 2 × 150 bp paired‐end (PE) configuration. Image analysis and base calling were conducted by the Control software. Raw sequence data (.bcl files) generated by the sequencer were converted into fastq files and demultiplexed using Illumina's bcl2fastq 2.17 software. One mismatch was allowed for index sequence identification.

#### 2.7.5 RNAseq data analysis

Sequence reads were trimmed to remove adapter sequences, and the trimmed reads were mapped to the reference genome available on ENSEMBL using the STAR aligner v.2.5.2b. BAM files were generated as a result of this step. Unique gene hit counts were calculated by using feature Counts from the Subread package v.1.5.2. Only unique reads that fell within exon regions were counted. After extraction of gene hit counts, the gene hit counts table was used for downstream analyses including a filter for calling likely expression. The expression filter was set to 10 cpm across replicate samples and genes were called as expressed if they were detected at ≥10 cpm in all replicates.

### 2.8 Statistical analysis

**FIGURE 2:**
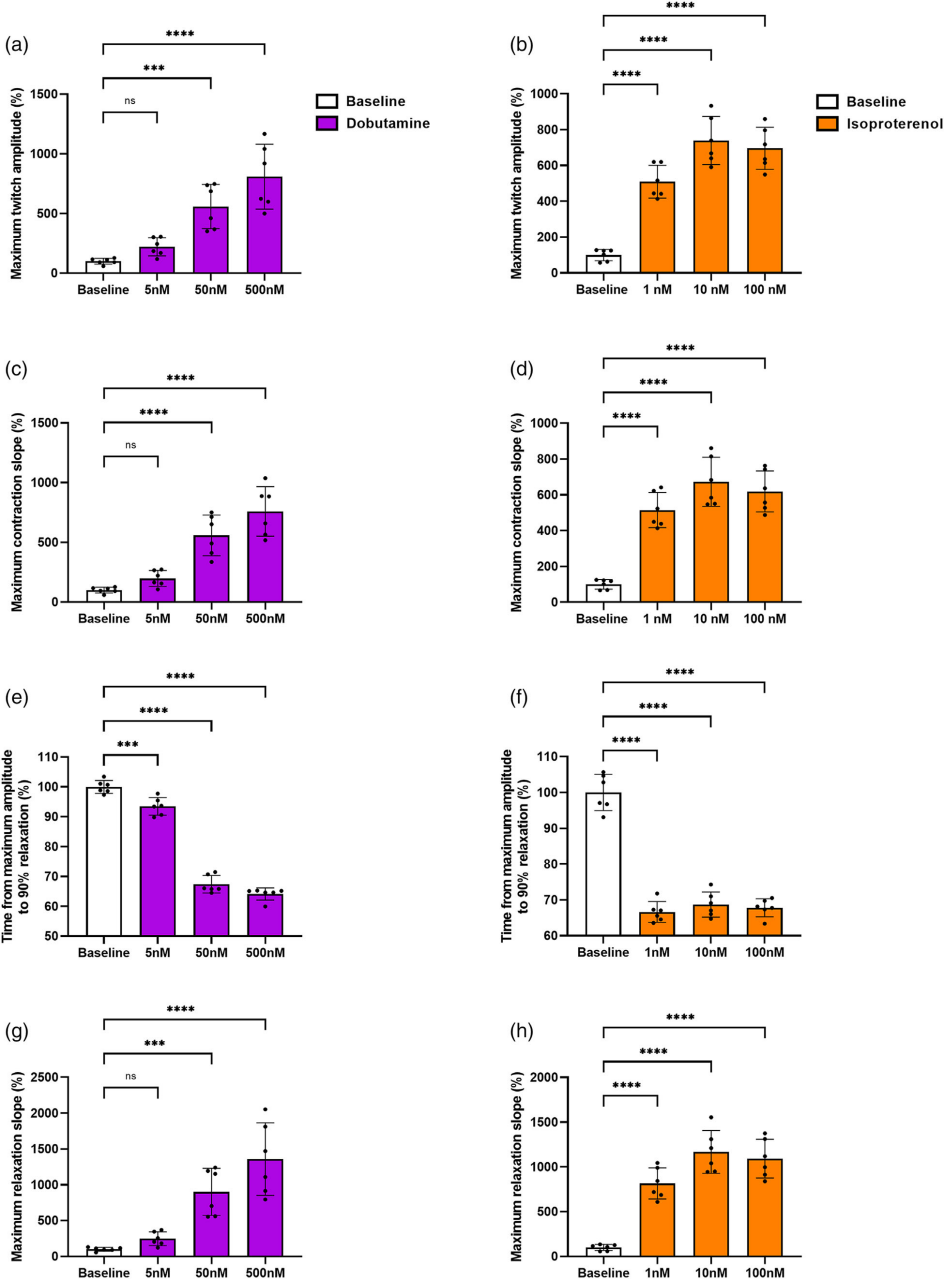
Isoproterenol and dobutamine treatment induce positive inotropic and lusitropic contractility responses in 3D‐engineered cardiac tissues. All data are expressed as a percentage of baseline. Values are means ± SD and represent *n* = 6, except (c) where *n* = 3. Significant difference between baseline and dobutamine/isoproterenol: ***P < 0.001, ****P < 0.0001.

**FIGURE 3:**
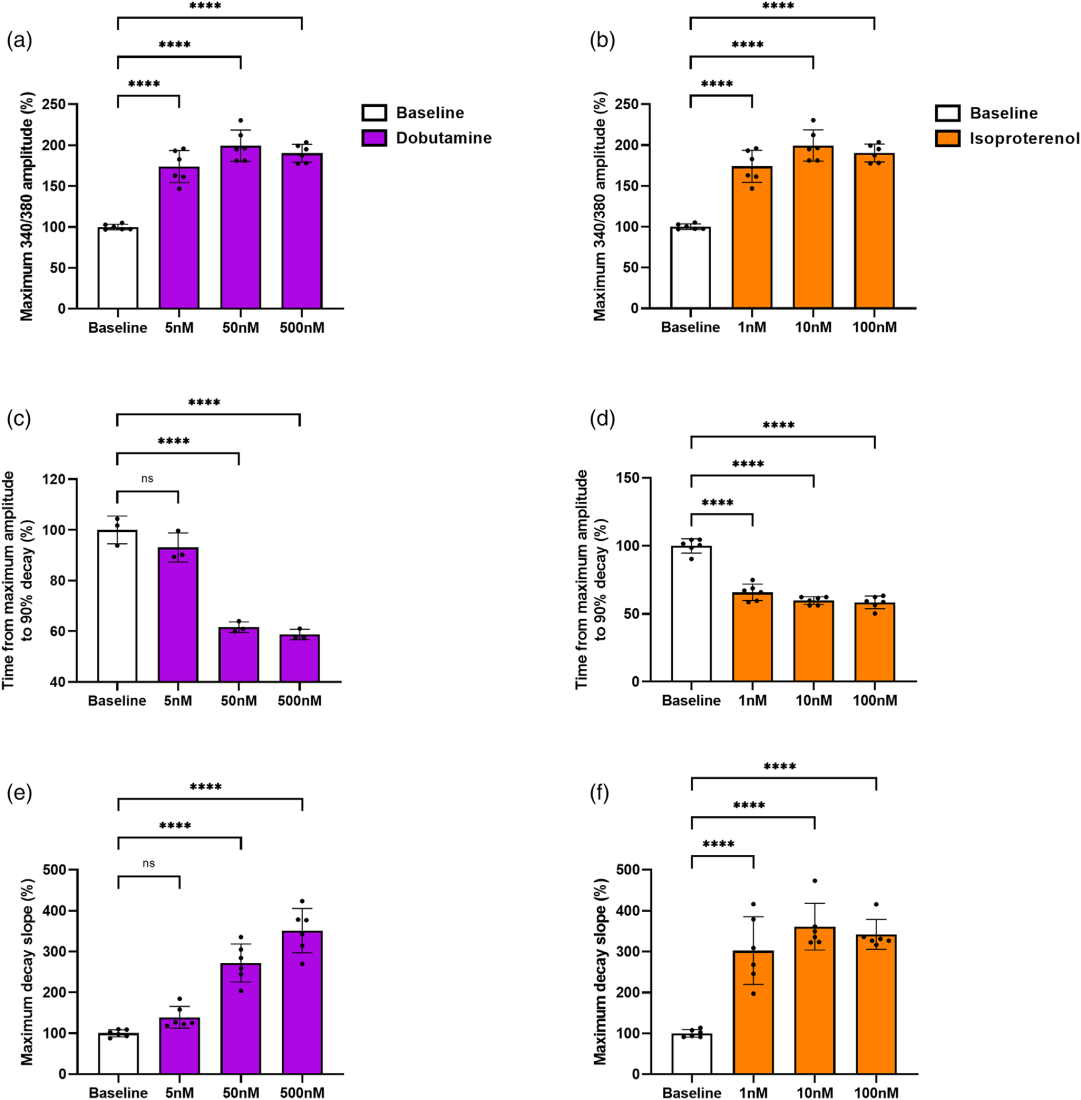
Isoproterenol and dobutamine treatment induce positive inotropic and lusitropic calcium responses in 3D‐engineered cardiac tissues. All data are expressed as a percentage of baseline. Values are means ± SD and represent *n* = 6. Significant difference between baseline and CNP: ****P < 0.0001. CNP, C‐type natriuretic peptide.

A Mann–Whitney test was used to detect differences in baseline values. For the *in vitro, ex vivo* and in vivo experiments, a two‐way ANOVA followed by Šídák's multiple comparison test was used, except for Figures 2 and 3, where a one‐way ANOVA with Dunnett's multiple comparisons test was used to test for differences from baseline. Analyses were performed separately for the period before and after ischaemia in the *ex vivo* experiments. All analysis were conducted in GraphPad Prism software (version 9.0.1, GraphPad Software, San Diego, CA, USA). Statistical significance was set at *P* < 0.05. Values are expressed as means ± SD.

## 3 RESULTS

### 3.1 RNA sequencing confirmed NPR2 expression and tissue maturity

RNA sequencing was performed on ECTs after maturation to confirm tissue maturity and CNP binding receptor expression (NPR2, NPR3). Gene expression profiles of *ATP2A2* (SERCA2), *SLC2A4* (glucose transporter type 4, GLUT4), *IGF1*, *IGF1R*, *INSR*, *NPPA* (atrial naturetic peptide, ANP), *NPPB* (brain natriuretic peptide, BNP), *NPPC* (C‐type natriuretic peptide, CNP), *NPR2*, *NPR3*, *PRKG1*, *PRKG2*, *RYR2* and *TNNI3* were assessed. All selected markers were expressed above threshold, except for the mRNA levels of *IGF1* and *NPPC*. Similar to that observed in cardiomyocytes and fibroblasts from human cardiac tissues (Koenig et al., 2022), *NPR2* was also highly expressed in the ECTs.

### 3.2 In vitro effects of dobutamine and isoproterenol in 3D‐engineered cardiac tissues

In a separate experiment to demonstrate responsiveness to known β‐adrenergic drugs, ECTs was exposed to increasing concentrations of dobutamine (5–500 nM) and isoproterenol (1–100 nM). Both drugs caused dose‐dependent increases in inotropic and lusitropic responses of ECTs (Figures 2 and 3).

Dobutamine (5 nM: *P* = 0.4793, 50 nM: *P* = 0.0004, 500 nM: *P* < 0.0001) and isoproterenol (1 nM: *P* < 0.0001, 10 nM: *P* < 0.0001, 100 nM: *P* < 0.0001) increased the maximum twitch amplitude (Figure 2a,b). Similarly, dobutamine (5 nM: *P* = 0.4894, 50 nM: *P* < 0.0001, 500 nM: *P* < 0.0001) and isoproterenol (1 nM: *P* < 0.0001, 10 nM: *P* < 0.0001, 100 nM: *P* < 0.0001) increased the maximum contraction slope (Figure 2c,d). These changes were accompanied by increased maximum calcium amplitude (340/380 ratio) for both drugs (*P* < 0.0001) (Figure 3a,b).

Both dobutamine (5 nM: *P* = 0.0008, 50 nM: *P* < 0.0001, 500 nM: *P* < 0.0001) and isoproterenol (1 nM: *P* < 0.0001, 10 nM: *P* < 0.0001, 100 nM: *P* < 0.0001) decreased the time from maximum amplitude to 90% relaxation (Figure 2e,f), which was accompanied by an increased maximum relaxation slope for both dobutamine (5 nM: *P* = 0.7450, 50 nM: *P* = 0.0006, 500 nM: *P* < 0.0001) and isoproterenol (1 nM: *P* < 0.0001, 10 nM: *P* < 0.0001, 100 nM: *P* < 0.0001) (Figure 2g,h). This was also evident on calcium kinetics, where the time from maximum amplitude to 90% decay was decreased (*P* < 0.0001) with both drugs, except for 5 nM dobutamine (*P* = 0.1795) (Figure 3c,d). Also, the maximum decay slope was increased with both agonists (*P* < 0.0001), apart from 5 nM dobutamine (*P* = 0.2169) (Figure 3e,f).

### 3.3 In vitro effects of CNP application in 3D‐engineered cardiac tissues

After 7 weeks of maturation, the ECTs reached an average force of ∼13 μN, and baseline parameters of contractility and calcium transients were similar between groups, except for calcium ratio (340/380) time to decay at 90% (s), which was higher in the CNP group compared to the vehicle group (0.36 vs. 0.34 s, respectively, *P* < 0.0206; Table 1).

**TABLE 1:** In vitro contractility and calcium parameters at baseline.

	Vehicle	CNP	*P*
Contractility			
Twitch amplitude (μN)	12.88 ± 6.40	13.55 ± 3.12	0.662
Maximum contraction slope (μN/s)	246.07 ± 117.11	261.70 ± 64.31	0.573
Time to relaxation 90% (s)	0.16 ± 0.01	0.17 ± 0.01	0.296
Maximum relaxation slope (μN/s)	−107.36 ± 56.74	−108.27 ± 25.48	0.755
Calcium			
340/380 (ratio)	0.14 ± 0.01	0.14 ± 0.01	0.471
Time to decay 90% (s)	0.34 ± 0.02	0.36 ± 0.01	0.021*
Maximum decay slope (ratio/s)	−0.51 ± 0.03	−0.47 ± 0.03	0.063

*Note*: Significant difference between vehicle and CNP at baseline: *P < 0.05.

**FIGURE 4:**
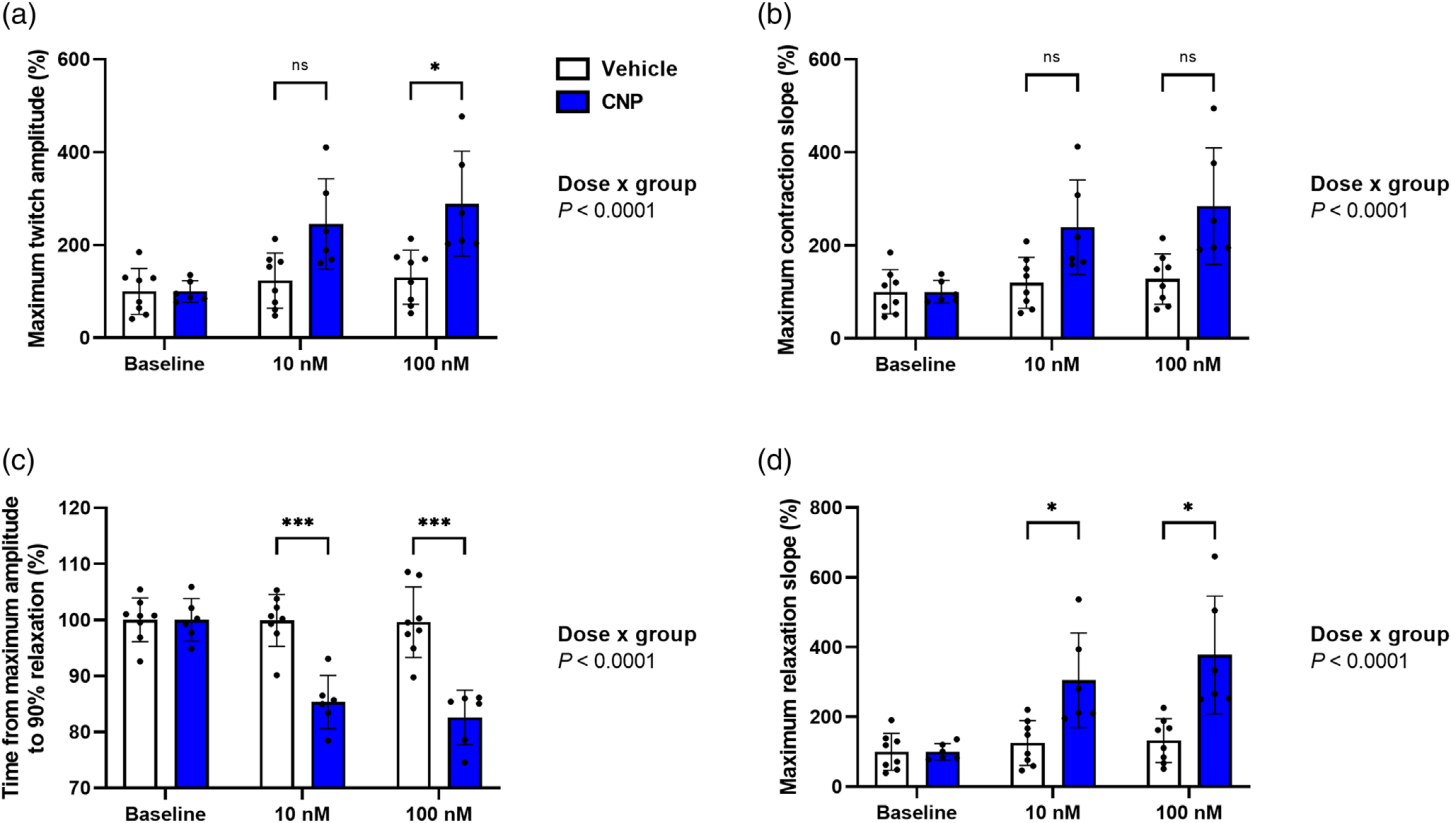
CNP treatment induces positive inotropic and lusitropic contractility responses in 3D‐engineered cardiac tissues. All data are expressed as a percentage of their own baseline. Values are means ± SD and represent *n* = 8 (vehicle) and *n* = 6 (CNP). Significant difference between vehicle and CNP: *P < 0.05, ***P < 0.001. CNP, C‐type natriuretic peptide; ISO, isoproterenol.

**FIGURE 5:**
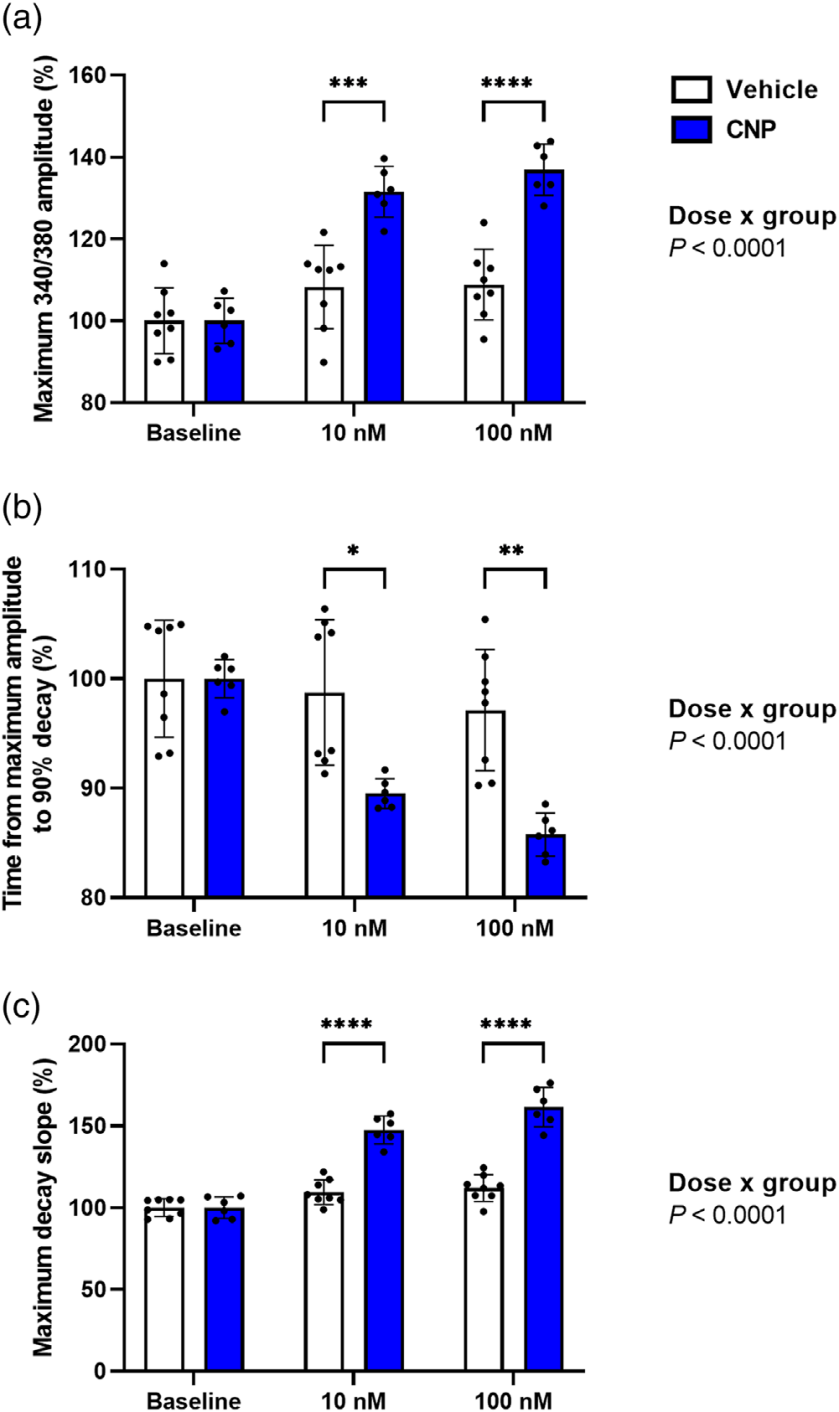
CNP treatment induces positive inotropic and lusitropic calcium responses in 3D‐engineered cardiac tissues. All data are expressed as a percentage of their own baseline. Values are means ± SD and represent *n* = 8 (vehicle) and *n* = 6 (CNP). Significant difference between vehicle and CNP: *P < 0.05, **P < 0.01, ***P < 0.001, ****P < 0.0001. CNP, C‐type natriuretic peptide; ISO, isoproterenol.

Application of CNP (10 and 100 nM) elicited a positive inotropic response (dose × group, *P* < 0.0001, Figures 4a,b and 5a). Specifically, CNP treatment increased maximum twitch amplitude (μN) as evidenced by a significant dose × group difference (*P* < 0.0001, Figure 4a). For individual comparisons, 10 nM CNP (*P* = 0.0549) did not reach statistical significance, whereas 100 nM CNP (*P* = 0.0489) was significantly increased compared to vehicle (Figure 4a). Similarly, CNP treatment induced an increase in the maximum contraction slope (μN/s) also evidenced by a significant dose × group difference (*P* < 0.0001, Figure 4b); however, numerical differences did not reach significance when performing individual comparisons between groups: 10 nM CNP (*P* = 0.0774) or 100 nM CNP (*P* = 0.0774) (Figure 4b). Also, the maximum calcium amplitude (340/380 ratio) was elevated during CNP treatment (dose × group, *P* < 0.0001, Figure 5a). Here, both 10 nM CNP (*P* = 0.0004) and 100 nM CNP (*P* < 0.0001) induced an increase (Figure 5a).

Acute CNP treatment also induced a positive lusitropic response (dose × group, *P* < 0.0001, Figures 4c,d and 5b,c). This was evidenced by reductions in time from peak contraction to 90% of relaxation (s) at both 10 nM CNP (*P* = 0.0003) and 100 nM CNP (*P* = 0.0003) (Figure 4c) and an increase in the maximum relaxation slope (μN/s) at both 10 nM CNP (*P* = 0.0427) and 100 nM CNP (*P* = 0.0427) (Figure 4d). These effects were paralleled by reductions in time from maximum calcium amplitude to 90% of decay (s) at both 10 nM CNP (*P* = 0.0106) and 100 nM CNP (*P* = 0.0013) and an increase in the maximum calcium decay slope (ratio/s) at 10 nM CNP (*P* < 0.0001) and 100 nM CNP (*P* < 0.0001) (Figure 5b,c).

Higher concentrations of CNP (300 nM and 1 μM) were also tested acutely in ECTs. Here, no differences were observed at baseline in ECTs between vehicle and CNP groups. Higher concentrations of CNP resulted in a similar inotropic and lusitropic response (dose × group, *P* < 0.0001) as those observed with the lower concentrations of CNP. High concentrations of CNP induced positive inotropic effects marked by an increased maximum twitch amplitude (300 nM and 1 μM; both *P* = 0.0210), maximum calcium amplitude (300 nM and 1 μM; both *P* < 0.0001), and maximum contraction slope (300 nM and 1 μM; both *P* = 0.0199). At high concentrations, CNP also elicited positive lusitropic responses demonstrated by decreased time from maximum amplitude to 90% relaxation (300 nM and 1 μM; both *P* < 0.0001) and time from maximum amplitude to 90% decay (300 nM and 1 μM; both *P* < 0.0001) followed by an increase in maximum relaxation slope (300 nM and 1 μM; both *P* = 0.0183) and maximum decay slope (300 nM and 1 μM; both *P* < 0.0001).

### 3.4 *Ex vivo* effects of CNP on rat cardiac function

**FIGURE 6:**
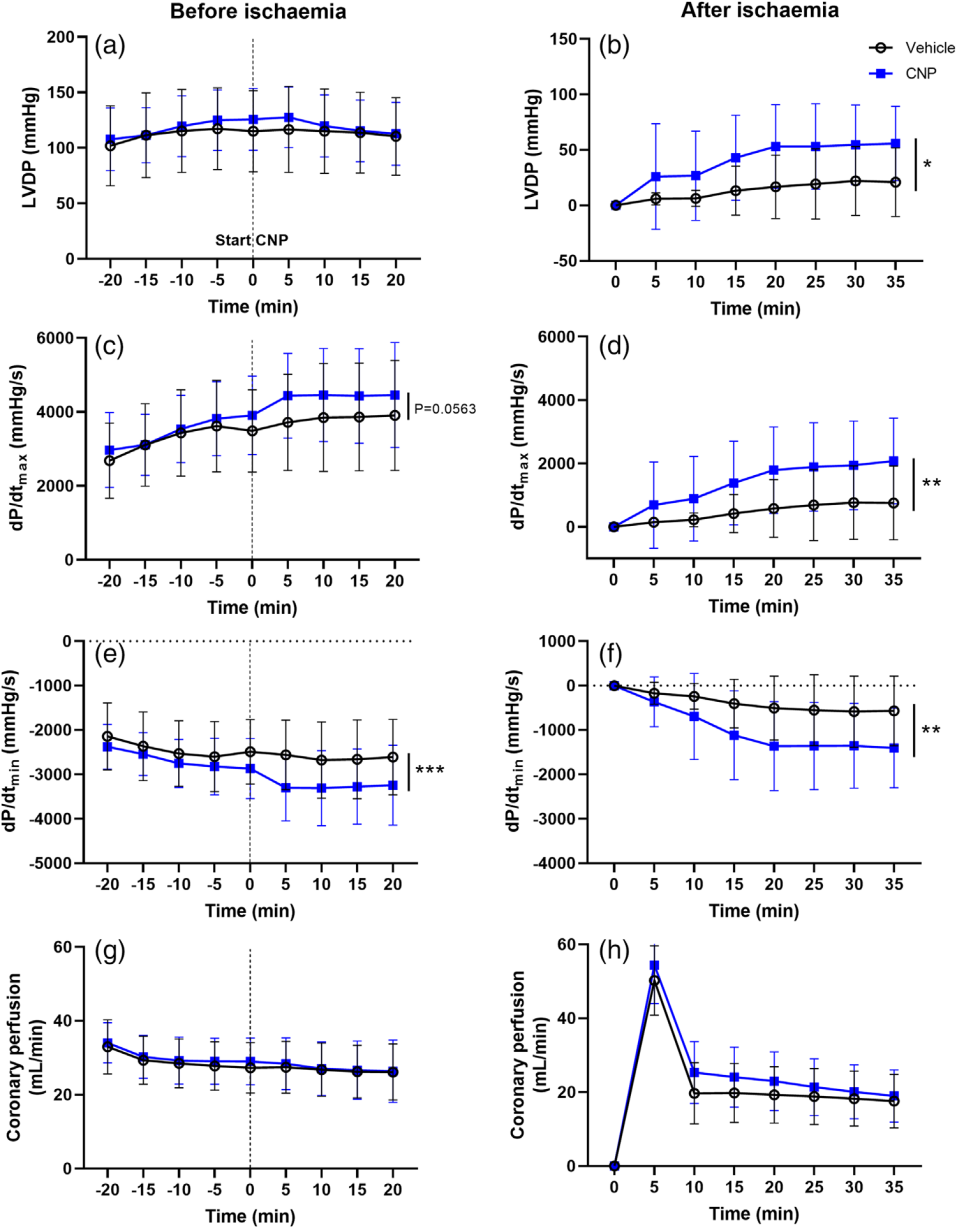
CNP treatment increases systolic and diastolic parameters in isolated hearts. Values are means ± SD and represent *n* = 14 (vehicle) and *n* = 15 (CNP). CNP was dissolved in Krebs–Henseleit buffer to a final concentration of 30 nM. Coronary perfusion was before and after 17.5 min of global ischaemia. Significant difference between vehicle and CNP: *P < 0.05, **P < 0.01, ***P < 0.001. CNP, C‐type natriuretic peptide; d*P*/d*t*_max_ and d*P*/d*t*_min_, maximal slope of the systolic pressure increment, and the diastolic pressure decrement; LVDP, left ventricular developed pressure.

In the first part of the Langendorff experiments, a suitable IR injury was established by testing IR periods of 15, 17.5, 20, 25 and 30 min. Ischaemic times of 20, 25 and 30 min resulted in large ~80% reductions in LVDP. Ischaemic time of 15 min resulted in no IR injury, whereas 17.5 min resulted in a ~45% reduction in LVDP. Prior to ischaemia in the Langendorff isolated heart model, no effect on LVDP was observed with CNP treatment (Figure 6a: individual data for all measurements included in Supplementary File 1). In contrast, CNP increased LVDP (35 min: ∼175%) following ischaemia (time × group, *P* = 0.0180, Figure 6b). There was a tendency towards an increase (*P* = 0.0563) in d*P*/d*t*_max_ with CNP prior to ischaemia (Figure 6c) that became significant (35 min: ∼175 %; time × group, *P* = 0.0053, Figure 6d) following the ischaemic insult. CNP treatment enhanced d*P*/d*t*_min_ both before (20 min: ∼25%, time × group, *P* = 0.0001, Figure 6e) and after (35 min: ∼150%, time × group, *P* = 0.0016, Figure 6f) ischaemia. Coronary perfusion was unaltered before and after ischaemia between groups (Figure 6g,h).

### 3.5 in vivo effects of CNP infusion on rat cardiac function

**FIGURE 7:**
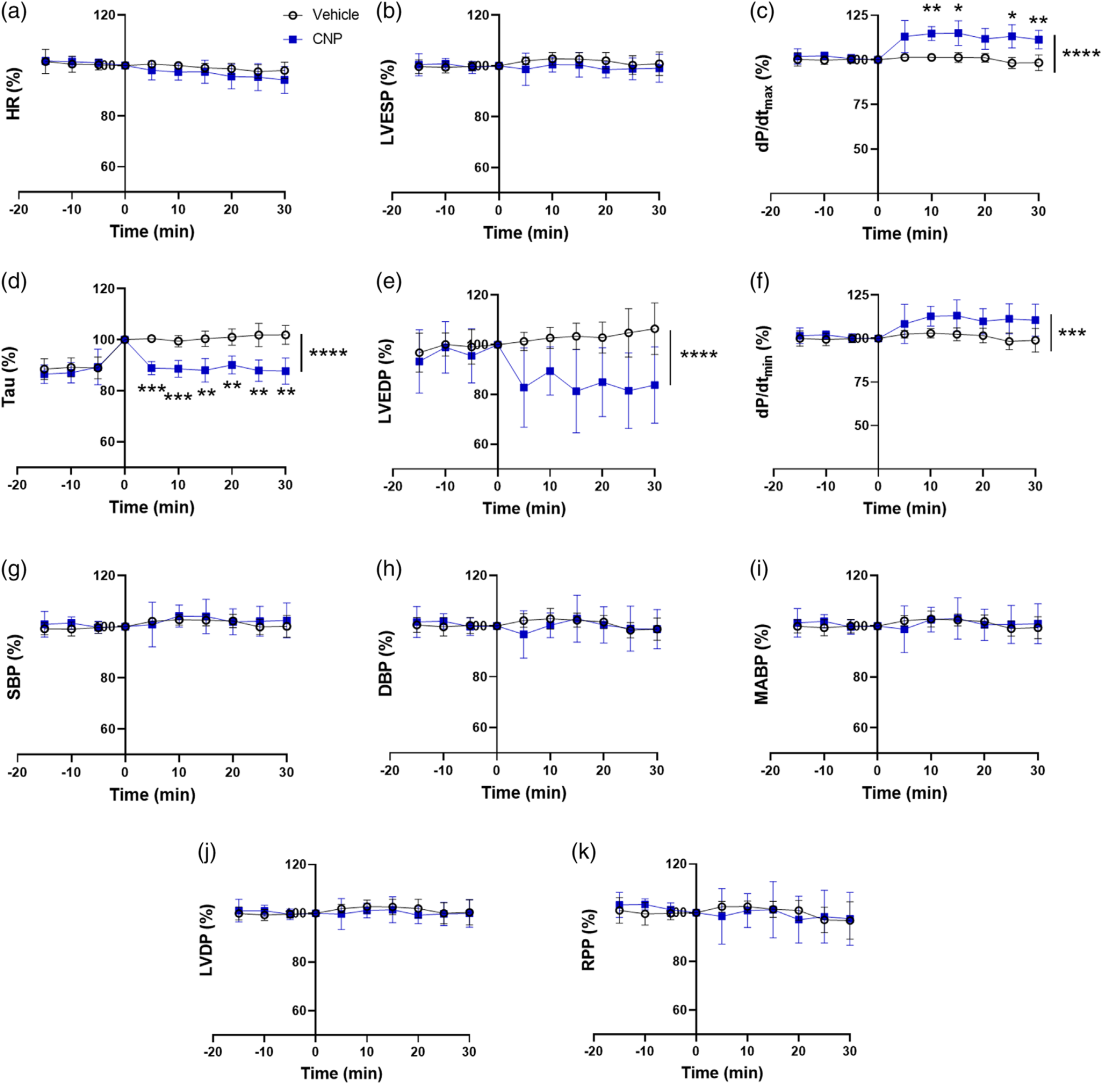
CNP infusion increases indices of cardiac performance in vivo without altering systemic hemodynamics. All data are expressed as a percentage of baseline (*t* = 0). Values are means ± SD and represent *n* = 7 (vehicle) and *n* = 6 (CNP). CNP was infused at a constant rate of 200 pmol/min/kg (plasma concentration of ∼10 nmol/l at steady state) via the tail vein. Significant difference between vehicle and CNP: ***P < 0.001, ****P < 0.0001. CNP, C‐type natriuretic peptide; DBP, diastolic blood pressure; d*P*/d*t*_max_ and d*P*/d*t*_min_, maximal slope of the systolic pressure increment, and the diastolic pressure decrement; HR, heart rate; LVDP, left ventricular developed pressure; LVEDP, left ventricular end‐diastolic pressure; LVESP, left ventricular end‐systolic pressure; MABP, mean arterial blood pressure; RPP, rate‐pressure product; SBP, systolic blood pressure.

There were no differences in indices of cardiac function and central hemodynamics at baseline (*t* = 0, Table 2: individual data for all measurements included in Supplementary File 2). Intravenous constant infusion of CNP improved cardiac contractility as evidenced by an increase (time × group, *P* < 0.0001) in d*P*/d*t*_max_ (Figure 7c). Similarly, diastolic function was enhanced as demonstrated by an increase (time × group, *P* = 0.0005) in d*P*/d*t*_min_ (Figure 7f) and lower (time × group, *P* < 0.0001) diastolic time constant Tau and LVEDP (compared to vehicle, Figure 7d,e). These cardiac effects of CNP infusion were observed in the absence of alterations in systolic blood pressure, diastolic blood pressure, mean arterial blood pressure, HR, LVESP, LVDP and RPP (Figure 7a,b,g–k).

**TABLE 2:** In vivo haemodynamic parameters at baseline (time = 0).

	Vehicle	CNP	*P*
HR (beats/min)	364 ± 11	374 ± 11	0.534
LVESP (mmHg)	109 ± 3	109 ± 2	>0.999
d*P*/d*t*_max_ (mmHg/s)	6494 ± 263	6670 ± 160	0.731
Tau (ms)	12 ± 1	10 ± 1	0.101
LVEDP (mmHg)	11 ± 1	9 ± 2	>0.999
d*P*/d*t*_min_ (mmHg/s)	−5530 ± 311	−5736 ± 115	0.836
SBP (mmHg)	113 ± 5	118 ± 3	0.534
DBP (mmHg)	73 ± 3	75 ± 2	0.731
MABP (mmHg)	87 ± 4	90 ± 2	0.732
LVDP (mmHg)	97 ± 4	100 ± 1	0.836
RPP (beats/min/mmHg)	41,505 ± 2931	44,334 ± 1426	0.366

Abbreviations: DBP, diastolic blood pressure; d*P*/d*t*_max_ and d*P*/d*t*_min_, maximal slope of the systolic pressure increment, and the diastolic pressure decrement; HR, heart rate; LVDP, left ventricular developed pressure; LVEDP, left ventricular end‐diastolic pressure; LVESP, left ventricular end‐systolic pressure; MABP, mean arterial blood pressure; RPP, rate pressure product; SBP, systolic blood pressure.

## 4 DISCUSSION

By utilizing a human ECT model, we demonstrate for the first time that CNP induces an inotropic and lusitropic response in a 3D in vitro system composed of matured human iPSC‐derived cardiomyocytes. The effects on kinetics mirrored those observed in rat *ex vivo* and in vivo model systems, whereas an increase in maximal force generation with CNP application was only observed in ECTs.

In the human heart, CNP is both produced and released to the circulation as evidenced by positive arteriovenous gradients for CNP and N‐terminal pro C‐type natriuretic peptide (NT‐proCNP) across the working heart (Palmer et al., 2009). The cellular source of CNP is likely to include endothelial cells, cardiac fibroblasts and cardiomyocytes as CNP is expressed in these cell types in the human heart (Koenig et al., 2022). Once released from the cell, CNP may induce paracrine and autocrine signalling via NPR2 expressed by cardiomyocytes (Koenig et al., 2022; Subramanian et al., 2018) where the present findings in ECTs suggest that it will have a modulatory effect on cardiac contractility. This proposed translatability of acute effects of CNP from ECTs into humans is supported by findings of canonical responses to a variety of small molecule modulators of cardiac contractility and calcium kinetics at pharmacologically relevant concentrations in similar ECTs (Feric et al., 2019).

To further establish the physiological relevance of the observed effects of CNP in ECTs, CNP‐induced changes in contractility were assessed in rat *ex vivo* and in vivo models in which circulating concentrations of CNP were matched to those applied in ECTs. In both the isolated heart preparation and in the intact animals, increases in d*P*/d*t*_max_ and d*P*/d*t*_min_ (as well as lower tau and LVEDP in vivo) were detected following CNP stimulation, thus mirroring the effects on contractility and calcium kinetics observed in ECTs. The only exception here was the lack of significance with regards to change in d*P*/d*t*_max_ in the *ex vivo* model; however, this is likely to reflect insufficient power to detect changes in this specific variable given the effect size of ∼20% and very low *P*‐value of 0.063. This translatability between model systems, which to some extent may reflect a very high conservation within the CNP system (Potter et al., 2009), indicates that other findings on the cardiac effects of CNP signalling in preclinical models may also translate very well.

We did observe one discrepancy between the models wherein an increase in developed force (twitch amplitude) was only detected in ECTs while LVDP remains unaltered in the intact heart (excluding the effect observed following ischaemia). This lack of increase in inotropy could, however, reflect that the *ex vivo* and in vivo setting provides an integrative response to CNP stimulation in which afterload (arterial blood pressure) is an important physiological determinant of LVDP. Hence, as blood pressure was not altered in these models, it may be speculated that a CNP‐induced increase in developed pressure may be evident in situations where arterial blood pressure is increased. In support of this proposition, a positive correlation between coronary sinus plasma CNP concentrations and mean pulmonary artery pressure has been reported in humans (Palmer et al., 2009).

Regarding the finding of unaltered blood pressure dynamics in the in vivo model, it should be noted that blood pressure is the product of cardiac output and systemic vascular resistance. Hence, a potential augmentation of blood pressure as a result of an increase in cardiac output may have been counterbalanced by a CNP‐induced reduction in systemic vascular resistance (peripheral vasodilatation). However, the finding that LVDP and a proxy for cardiac work (RPP) did not change with treatment supports that CNP is not acting as an inotropic agent that increases cardiac output. Hence, the unaltered blood pressure is likely to reflect that CNP alters cardiac kinetics without altering stroke volume and systemic resistance in these healthy animals.

Early studies using isolated right atria preparations from healthy dogs have reported positive inotropic (Beaulieu et al., 1997; Hirose et al., 1998) and lusitropic (Beaulieu et al., 1997) responses to CNP application. However, more recent studies in rodent HF models examining the effects of CNP on isolated cardiac muscle strips have demonstrated negative inotropic and positive lusitropic effects (Manfra et al., 2022; Moltzau et al., 2013, 2014; Qvigstad et al., 2010). Yet others have reported a positive acute inotropic response followed by a negative inotropic response in wild‐type (WT) isolated mouse heart preparations (Pierkes et al., 2002; Wollert et al., 2003). Taken together, these observations could indicate distinct effects of CNP depending on the health status of the tissue (Table 3). The experiments in the present study were performed in healthy tissue preparations, and the findings are thus in line with previous findings on CNP in healthy or WT tissue preparations, although we were not able to detect a potential decline in developed force given the single sampling point. Importantly, future studies should assess whether CNP induces negative inotropic responses in ECTs displaying a disease‐relevant phenotype.

**TABLE 3:** Overview of in vitro, *ex vivo* and in vivo findings of CNP effects on cardiac function.

Study	System	Species	Parameter measured (among others)	CNP effect	Disease state
In vitro					
Current study	ECTs	Human	Maximum twitch amplitude (μN); maximum contraction/relaxation slope (μN/s); time to peak/relaxation (s). Calcium transient amplitude (*F*/*F*_0_ ratio) time to pick/decay (s), maximum decay slope (ratio/s)	Positive inotropic and lusitropic response (10–100 nM, 300 nM–1 μM)	Healthy
Cachorro et al. (2023)	Isolated CMs	Mouse	FS (%) Contraction/relaxation velocity (μm/μs)	Neutral effect on FS; increased contraction velocity Positive lusitropic effect (1 μM)	Healthy
Szaroszyk et al. (2022)	Isolated CMs	Mouse	Cell shortening (%) Calcium transient amplitude (*F*/*F*_0_ ratio)	Positive inotropic effect Increased Ca^2+^ transient amplitude (10–100 nM)	Healthy
Moltzau et al. (2013, 2014)	Isolated CMs	Rat	Calcium transient amplitude (*F*/*F*_0_ ratio)	Increase Ca^2+^ amplitude (biphasic response) (300 nM)	MI
Wollert et al. (2003)	Isolated CMs	Mouse	Cell shortening (%); time to 90% relaxation Calcium transient amplitude (*F*/*F*_0_ ratio); time to 50% decay	Increased Ca^2+^ transient amplitude (300 nM)	Healthy (WT and PKG TG mice)
*Ex vivo*					
Current study	Langendorff isolated heart model	Rat	LVDP, d*P*/d*t*_min/max_ (mmHg/s)	Positive inotropic and lusitropic response after ischaemia Positive lusitropic response before ischaemia (30 nM)	Baseline and after ischaemia
Manfra et al. (2022)	LV muscle strips	Rat	Maximum development of force (Δ*F*/d*t*_max_), time to peak force (TPF), and relaxation time (ΔRT = TR80 − TPF)	Negative ionotropic response Positive lusitropic response (concentration–response curves)	MI
Moltzau et al. (2013, 2014)	LV muscle strips	Rat	Maximum development of force (Δ*F*/d*t*_max_), time to peak, time to 80% relaxation (ms), and relaxation time (ΔRT)	Negative ionotropic response Positive lusitropic response (concentration–response curves)	MI and sham
Qvigstad et al. (2010)	LV muscle strips	Rat	Contractile force *F*_max_ (mN)	Negative inotropic response but enhanced β_1_‐adrenergic‐mediated inotropic effect (concentration response curves/300 nM)	MI
Wollert et al. (2003)	Isolated perfused working heart	Mouse	±d*P*/d*t* (mmHg/s) Time to peak; time to relaxation (ms)	Positive inotropic and lusitropic effect (10 nM)	Healthy (WT and PKG TG mice)
Pierkes et al. (2002)	Isolated perfused working heart	Mouse	±d*P*/d*t* (mmHg/s) Time to peak; time to relaxation (ms)	Positive ionotropic and lusitropic followed by negative ionotropic response (biphasic response) (1–100 nM)	Healthy (WT and GC‐A‐deficient)
Hirose et al. (1998)	Atrial and LV preparation	Dog	Contractile force (g)	Positive inotropic response (0.1–0.3 nM)	Healthy
Beaulieu et al. (1997)	Isolated atrial preparation	Dog	Contractile force (g)	Positive inotropic response (25 μg/0.5 mL/1 min injected in SAN artery). Indirect positive lusitropic effect by increased maximal rate of diastolic depolarization and decreased AP time	Healthy
in vivo					
Current study	in vivo (haemodynamics)	Mouse	LVESP, LVDP (mmHg), d*P*/d*t*_max_ and d*P*/d*t*_min_ (mmHg/s), diastolic time constant tau, and LVEDP	Positive ‘inotropic’ (with no change in LVDP) and lusitropic effect (200 pmol/min/kg constant i.v. infusion)	Healthy
Soeki et al. (2005)	in vivo (haemodynamics and echocardiography)	Rat	LVEDP (mmHg), d*P*/d*t*_max_ and d*P*/d*t*_min_ (mmHg/s), cardiac output (ml/min), FS (%), *E*/*A*	Positive inotropic and lusitropic effects (osmotic minipump: 0.1 μg/kg/min – 2 weeks)	MI
Izumiya et al. (2012)	in vivo (echocardiography)	Mouse	PWT, LVEDD, FS (%)	Neutral effect on healthy animal, attenuation of AngII‐induced FS decrease (osmotic minipump: 0.05 μg/kg/min − 2 weeks)	AngII infusion model
Li et al. (2016)	in vivo (haemodynamics)	Dog	LVEDP, LVESP, LVEDV, d*P*/d*t*_max_ and d*P*/d*t*_min_ (mmHg/s), cardiac output (ml/min) (among others)	Positive inotropic and lusitropic response (2 mg/kg + 0.4 mg/kg/min, i.v., 20 min)	Paced‐induced HF

Abbreviations: AngII, angiotensin II; AP, action potential; CM, cardiomyocyte; *E*/*A*, atrial filling wave; *E*, early filling wave; ECT, engineered cardiac tissue; FS, fractional shortening; GC‐A, guanylyl cyclase‐A; LV, left ventricular; LVEDD, left ventricular end‐diastolic; LVEDP, left ventricular end‐diastolic pressure; MI, myocardial infarction; PKG, protein kinase G; PWT, posterior wall thickness; SAN, sinoatrial node; TG, transgenic; TR80, time to 80% relaxation; WT, wildtype.

In isolated cardiomyocytes, findings have been more varied when it comes to inotropic responses. In line with *ex vivo* findings, negative inotropic and positive lusitropic responses in isolated rat cardiomyocytes have been reported (Moltzau et al., 2013). In contrast, Wollert et al. (2003) showed increased cell shortening of isolated WT cardiomyocytes treated with 300 nM CNP along with decreased time to 90% relaxation. In another study, positive inotropic effects at 10 and 100 nM of CNP treatment in isolated mouse WT cardiomyocytes were reported (Szaroszyk et al., 2022). These results are in line with the observations in the present study, demonstrating positive inotropic and lusitropic effects at 10 and 100 nM of CNP in ECTs. In a recent study, CNP was shown to have antiarrhythmic effects in isolated murine cardiomyocytes, Langendorff‐perfused hearts and human iPSC‐derived cardiomyocytes (Cachorro et al., 2023). Although CNP promoted a lusitropic response, no changes in inotropy were observed at 1 μM CNP in their preparations of isolated murine cardiomyocytes. This study also examined the effects of CNP on cardiac parameters using human cells in vitro; however, contractility parameters were not assessed for the 2D hiPSC‐derived cardiomyocytes. Furthermore, calcium sparks detected in hiPSC‐derived cardiomyocytes were performed in a traditional 2D setting, which is associated with less mature cardiomyocytes compared to those in 3D ECTs. To our knowledge, no studies have examined the effects of CNP in isolated human cardiomyocytes. However, the use of isolated human cardiomyocytes faces major challenges related to the limited availability of human donor hearts, the unstable isolation efficiency and quality, and rapid cell death. In addition, single‐cell cardiomyocytes lack important mechanical properties such as stretch and loading present in cardiac tissues. In contrast, ECTs use human pluripotent stem‐cell derived cardiomyocytes that are readily available and can be cultured for long periods. Furthermore, the ECTs used in current study, exhibited high expression of the CNP binding receptors along with several markers of tissue maturity, although further investigation is required to determine protein levels. Future studies are needed to compare human‐isolated cardiomyocytes and ECTs to enhance our understanding of the translatability between these model systems.

Effects of CNP in vivo have been studied in several animal models of HF. Infusion of CNP in myocardial infarction (MI)‐induced rats increased LV d*P*/d*t*_max_ and fractional shortening, whereas LVEDP and LV d*P*/d*t*_min_ were decreased compared to the MI‐vehicle group, suggesting both positive inotropic and lusitropic effects of CNP (Soeki et al., 2005). In a mouse model of cardiac hypertrophy and remodelling induced by angiotensin II, CNP infusion also increased fractional shortening and decreased LVED dimension compared to the angiotensin II vehicle group, but no changes were observed with CNP when comparing with vehicle in the saline groups (Izumiya et al., 2012). In pacing‐induced HF in dogs, CNP augmented LV contraction, relaxation, diastolic filling and LV arterial coupling (Li et al., 2016). Taken collectively, observations in vivo suggest that CNP elicits both inotropic and lusitropic responses in preclinical models of HF.

One very consistent finding across model systems has been a CNP‐induced enhancement of diastolic function in both health and cardiac disease. When combined with the present observation of a similar lusitropic response to CNP application in ECTs and the findings of reduced CNP mRNA levels in the failing heart (Ichiki et al., 2014; Moyes et al., 2020), it may be speculated that a pharmacological treatment with a long‐acting CNP analogue could improve cardiac performance in patients suffering from impaired LV filling. Here, HF with preserved ejection fraction (HFpEF) would be one patient group in which such a pharmacotherapy could be effective, as one hallmark of HFpEF is impaired relaxation and/or increased viscoelastic chamber stiffness (Gaasch & Zile, 2004), leading to elevated filling pressures (Borlaug et al., 2016) that promote symptoms of dyspnoea, impair exercise capacity (Obokata et al., 2018), increase risk for HF hospitalization (Adamson et al., 2014), and decrease survival in HFpEF (Dorfs et al., 2014).

Some experimental considerations should be highlighted. Applied CNP concentrations *ex vivo* and in vivo were in the low nM range whereas CNP concentrations in human plasma have been reported to be in the low pM range (Palmer et al., 2009). Here, it should be emphasized that local concentrations of CNP in the contracting myocardium are expected to be significantly higher than what is observed in plasma since CNP is produced by cardiac tissue‐resident cells (Koenig et al., 2022), and any CNP that reaches the systemic circulation is diluted by the large plasma volume where it is also rapidly cleared (half‐life ∼2 min) by enzymatic degradation and NPR3‐mediated clearance (Potter et al., 2009). Interestingly, in ECTs, similar responses were observed with 10 and 100 nM CNP, which could indicate that a maximum effect was achieved at 10 nM. Thus, future studies should utilize lower concentrations to characterize a potential dose–response relationship in ECTs. Furthermore, to confirm the presence and functionality of pathways that modulate cardiac contractility in humans, ECTs were stimulated with isoproterenol and dobutamine and here canonical responses were observed. These confirmatory experiments were only performed in ECTs, as the cardiac responses to these pharmacological agents have been well‐described for both perfused heart preparations and in vivo settings (e.g., Dobson et al., 1990; Osadchii et al., 2007; Romano et al., 1991).

In conclusion, the present study demonstrates that CNP induces a positive inotropic and lusitropic response in human ECTs, thus supporting an important role for CNP in the regulation of human cardiac function. The finding that the in vitro effects of ECTs were highly reflective of CNP‐induced changes in rat cardiac dynamics *ex vivo* and in vivo provides additional support to this hypothesis and expands our current understanding of the translational value of ECTs. Future studies investigating the cardiac effects of manipulating CNP signalling in humans are warranted to substantiate the translational significance of the present findings in ECTs.

## AUTHOR CONTRIBUTIONS

Julian C. Bachmann, Jeppe E. Kirchhoff, Giulia Borghetti and Michael Nyberg conceived and designed the research. Jeppe E. Kirchhoff, Julia E. Napolitano, Steve Sorota, William M. Gordon, Nicole Feric, Roozbeh Aschar‐Sobbi, Juan Lv and Zhiyou Cao performed experiments and analysed data. Julian C. Bachmann, Jeppe E. Kirchhoff, Julia E. Napolitano, Steve Sorota, William M. Gordon, Nicole Feric, Roozbeh Aschar‐Sobbi, Giulia Borghetti and Michael Nyberg interpreted the results of experiments. Julian C. Bachmann and Michael Nyberg drafted the manuscript. Julian C. Bachmann, Jeppe E. Kirchhoff, Julia E. Napolitano, Steve Sorota, William M. Gordon, Nicole Feric, Roozbeh Aschar‐Sobbi, Ken Coppieters, Giulia Borghetti and Michael Nyberg edited and revised the manuscript. All authors have read and approved the final version of this manuscript and agree to be accountable for all aspects of the work in ensuring that questions related to the accuracy or integrity of any part of the work are appropriately investigated and resolved. All persons designated as authors qualify for authorship, and all those who qualify for authorship are listed.

## CONFLICT OF INTEREST

J.C.B., J.E.K., J.L., Z.C., K.C., G.B. and M.N. are employed by Novo Nordisk A/S. J.E.N., S.S., W.M.G., N.F. and R.A.‐S. are employed by Valo Health Inc.

## FUNDING INFORMATION

No funding was received for this work.

## Supporting information

Supplementary File 1

Supplementary File 2

## Data Availability

The datasets used and/or analysed during the present study are available from the corresponding author upon reasonable request.

## References

[eph13399-bib-0001] Adamson, P. B. , Abraham, W. T. , Bourge, R. C. , Costanzo, M. R. , Hasan, A. , Yadav, C. , Henderson, J. , Cowart, P. , & Stevenson, L. W. (2014). Wireless pulmonary artery pressure monitoring guides management to reduce decompensation in heart failure with preserved ejection fraction. Circulation Heart Failure, 7(6), 935–944. 25286913 10.1161/CIRCHEARTFAILURE.113.001229

[eph13399-bib-0002] Beaulieu, P. , Cardinal, R. , Page, P. , Francoeur, F. , Tremblay, J. , & Lambert, C. (1997). Positive chronotropic and inotropic effects of C‐type natriuretic peptide in dogs. American Journal of Physiology, 273, H1933–H1940. 9362263 10.1152/ajpheart.1997.273.4.H1933

[eph13399-bib-0003] Borlaug, B. A. , Kane, G. C. , Melenovsky, V. , & Olson, T. P. (2016). Abnormal right ventricular‐pulmonary artery coupling with exercise in heart failure with preserved ejection fraction. European Heart Journal, 37(43), 3293–3302. 10.1093/eurheartj/ehw241 PMC8483148 27354047

[eph13399-bib-0004] Botker, H. E. , Hausenloy, D. , Andreadou, I. , Antonucci, S. , Boengler, K. , Davidson, S. M. , Deshwal, S. , Devaux, Y. , Di Lisa, F. , Di Sante, M. , Efentakis, P. , Femmino, S. , Garcia‐Dorado, D. , Giricz, Z. , Ibanez, B. , Iliodromitis, E. , Kaludercic, N. , Kleinbongard, P. , Neuhauser, M. , … Heusch, G. (2018). Practical guidelines for rigor and reproducibility in preclinical and clinical studies on cardioprotection. Basic Research in Cardiology, 113(5), 39. 30120595 10.1007/s00395-018-0696-8 PMC6105267

[eph13399-bib-0005] Cachorro, E. , Gunscht, M. , Schubert, M. , Sadek, M. S. , Siegert, J. , Dutt, F. , Bauermeister, C. , Quickert, S. , Berning, H. , Nowakowski, F. , Lammle, S. , Firneburg, R. , Luo, X. , Kunzel, S. R. , Klapproth, E. , Mirtschink, P. , Mayr, M. , Dewenter, M. , Vettel, C. , … Kammerer, S. (2023). CNP promotes antiarrhythmic effects via phosphodiesterase 2. Circulation Research, 132(4), 400–414. 36715019 10.1161/CIRCRESAHA.122.322031 PMC9930893

[eph13399-bib-0006] Cerra, M. C. , & Pellegrino, D. (2007). Cardiovascular cGMP‐generating systems in physiological and pathological conditions. Current Medicinal Chemistry, 14(5), 585–599. 17346149 10.2174/092986707780059715

[eph13399-bib-0007] Dobson, J. G., Jr. , Fenton, R. A. , & Romano, F. D. (1990). Increased myocardial adenosine production and reduction of beta‐adrenergic contractile response in aged hearts. Circulation Research, 66(5), 1381–1390. 2159390 10.1161/01.res.66.5.1381

[eph13399-bib-0008] Dorfs, S. , Zeh, W. , Hochholzer, W. , Jander, N. , Kienzle, R. P. , Pieske, B. , & Neumann, F. J. (2014). Pulmonary capillary wedge pressure during exercise and long‐term mortality in patients with suspected heart failure with preserved ejection fraction. European Heart Journal, 35(44), 3103–3112. 25161181 10.1093/eurheartj/ehu315

[eph13399-bib-0009] Feric, N. T. , Pallotta, I. , Singh, R. , Bogdanowicz, D. R. , Gustilo, M. , Chaudhary, K. , Willette, R. N. , Chendrimada, T. , Xu, X. , Graziano, M. P. , & Aschar‐Sobbi, R. (2019). Engineered cardiac tissues generated in the biowire II: A platform for human‐based drug discovery. Toxicological Sciences, 172(1), 89–97. 31385592 10.1093/toxsci/kfz168 PMC6813749

[eph13399-bib-0010] Gaasch, W. H. , & Zile, M. R. (2004). Left ventricular diastolic dysfunction and diastolic heart failure. Annual Review of Medicine, 55(1), 373–394. 10.1146/annurev.med.55.091902.104417 14746527

[eph13399-bib-0011] Hirose, M. , Furukawa, Y. , Kurogouchi, F. , Nakajima, K. , Miyashita, Y. , & Chiba, S. (1998). C‐type natriuretic peptide increases myocardial contractility and sinus rate mediated by guanylyl cyclase‐linked natriuretic peptide receptors in isolated, blood‐perfused dog heart preparations. Journal of Pharmacology and Experimental Therapeutics, 286, 70–76. 9655843

[eph13399-bib-0012] Ichiki, T. , Schirger, J. A. , Huntley, B. K. , Brozovich, F. V. , Maleszewski, J. J. , Sandberg, S. M. , Sangaralingham, S. J. , Park, S. J. , & Burnett, J. C., Jr. (2014). Cardiac fibrosis in end‐stage human heart failure and the cardiac natriuretic peptide guanylyl cyclase system: Regulation and therapeutic implications. Journal of Molecular and Cellular Cardiology, 75, 199–205. 25117468 10.1016/j.yjmcc.2014.08.001 PMC4157955

[eph13399-bib-0013] Izumiya, Y. , Araki, S. , Usuku, H. , Rokutanda, T. , Hanatani, S. , & Ogawa, H. (2012). Chronic C‐type natriuretic peptide infusion attenuates angiotensin II‐induced myocardial superoxide production and cardiac remodeling. International Journal Vascular Medicine, 2012, 246058. 10.1155/2012/246058 PMC3405723 22848833

[eph13399-bib-0014] Koenig, A. L. , Shchukina, I. , Amrute, J. , Andhey, P. S. , Zaitsev, K. , Lai, L. , Bajpai, G. , Bredemeyer, A. , Smith, G. , Jones, C. , Terrebonne, E. , Rentschler, S. L. , Artyomov, M. N. , & Lavine, K. J. (2022). Single‐cell transcriptomics reveals cell‐type‐specific diversification in human heart failure. Nature Cardiovascular Research, 1(3), 263–280. 10.1038/s44161-022-00028-6 PMC9364913 35959412

[eph13399-bib-0015] Kusunose, K. , Penn, M. S. , Zhang, Y. , Cheng, Y. , Thomas, J. D. , Marwick, T. H. , & Popovic, Z. B. (2012). How similar are the mice to men? Between‐species comparison of left ventricular mechanics using strain imaging. PLoS ONE, 7(6), e40061. 22768220 10.1371/journal.pone.0040061 PMC3386935

[eph13399-bib-0016] Li, T. , Cheng, H. J. , Ohte, N. , Hasegawa, H. , Morimoto, A. , Herrington, D. M. , Little, W. C. , Li, W. , & Cheng, C. P. (2016). C‐type natriuretic peptide improves left ventricular functional performance at rest and restores normal exercise responses after heart failure. Journal of Pharmacology and Experimental Therapeutics, 357(3), 545–553. 27026682 10.1124/jpet.115.231696 PMC4885509

[eph13399-bib-0017] Manfra, O. , Calamera, G. , Froese, A. , Arunthavarajah, D. , Surdo, N. C. , Meier, S. , Melleby, A. O. , Aasrum, M. , Aronsen, J. M. , Nikolaev, V. O. , Zaccolo, M. , Moltzau, L. R. , Levy, F. O. , & Andressen, K. W. (2022). CNP regulates cardiac contractility and increases cGMP near both SERCA and TnI: Difference from BNP visualized by targeted cGMP biosensors. Cardiovascular Research, 118(6), 1506–1519. 33970224 10.1093/cvr/cvab167 PMC9074987

[eph13399-bib-0018] Milani‐Nejad, N. , & Janssen, P. M. (2014). Small and large animal models in cardiac contraction research: Advantages and disadvantages. Pharmacology & Therapeutics, 141(3), 235–249. 24140081 10.1016/j.pharmthera.2013.10.007 PMC3947198

[eph13399-bib-0019] Moltzau, L. R. , Aronsen, J. M. , Meier, S. , Nguyen, C. H. , Hougen, K. , Orstavik, O. , Sjaastad, I. , Christensen, G. , Skomedal, T. , Osnes, J. B. , Levy, F. O. , & Qvigstad, E. (2013). SERCA2 activity is involved in the CNP‐mediated functional responses in failing rat myocardium. British Journal of Pharmacology, 170(2), 366–379. 23808942 10.1111/bph.12282 PMC3834760

[eph13399-bib-0020] Moltzau, L. R. , Aronsen, J. M. , Meier, S. , Skogestad, J. , Orstavik, O. , Lothe, G. B. , Sjaastad, I. , Skomedal, T. , Osnes, J. B. , Levy, F. O. , & Qvigstad, E. (2014). Different compartmentation of responses to brain natriuretic peptide and C‐type natriuretic peptide in failing rat ventricle. Journal of Pharmacology and Experimental Therapeutics, 350(3), 681–690. 25022512 10.1124/jpet.114.214882

[eph13399-bib-0021] Moyes, A. J. , Chu, S. M. , Aubdool, A. A. , Dukinfield, M. S. , Margulies, K. B. , Bedi, K. C. , Hodivala‐Dilke, K. , Baliga, R. S. , & Hobbs, A. J. (2020). C‐type natriuretic peptide co‐ordinates cardiac structure and function. European Heart Journal, 41(9), 1006–1020. 30903134 10.1093/eurheartj/ehz093 PMC7068173

[eph13399-bib-0022] Moyes, A. J. , & Hobbs, A. J. (2019). C‐type natriuretic peptide: A multifaceted paracrine regulator in the heart and vasculature. International Journal of Molecular Sciences, 20(9), 2281. 31072047 10.3390/ijms20092281 PMC6539462

[eph13399-bib-0023] Nyberg, M. , Terzic, D. , Ludvigsen, T. P. , Mark, P. D. , Michaelsen, N. B. , Abildstrom, S. Z. , Engelmann, M. , Richards, A. M. , & Goetze, J. P. (2023). A state of natriuretic peptide deficiency. Endocrine Reviews, 44(3), 379–392. 36346821 10.1210/endrev/bnac029 PMC10166265

[eph13399-bib-0024] Obokata, M. , Olson, T. P. , Reddy, Y. N. V. , Melenovsky, V. , Kane, G. C. , & Borlaug, B. A. (2018). Haemodynamics, dyspnoea, and pulmonary reserve in heart failure with preserved ejection fraction. European Heart Journal, 39(30), 2810–2821. 29788047 10.1093/eurheartj/ehy268 PMC6658816

[eph13399-bib-0025] Osadchii, O. , Norton, G. , Deftereos, D. , & Woodiwiss, A. (2007). Rat strain‐related differences in myocardial adrenergic tone and the impact on cardiac fibrosis, adrenergic responsiveness and myocardial structure and function. Pharmacological Research, 55(4), 287–294. 17257851 10.1016/j.phrs.2006.12.005

[eph13399-bib-0026] Palmer, S. C. , Prickett, T. C. , Espiner, E. A. , Yandle, T. G. , & Richards, A. M. (2009). Regional release and clearance of C‐type natriuretic peptides in the human circulation and relation to cardiac function. Hypertension, 54(3), 612–618. 19620509 10.1161/HYPERTENSIONAHA.109.135608

[eph13399-bib-0027] Pierkes, M. , Gambaryan, S. , Boknik, P. , Lohmann, S. M. , Schmitz, W. , Potthast, R. , Holtwick, R. , & Kuhn, M. (2002). Increased effects of C‐type natriuretic peptide on cardiac ventricular contractility and relaxation in guanylyl cyclase A‐deficient mice. Cardiovascular Research, 53(4), 852–861. 11922895 10.1016/s0008-6363(01)00543-0

[eph13399-bib-0028] Popovic, Z. B. , Sun, J. P. , Yamada, H. , Drinko, J. , Mauer, K. , Greenberg, N. L. , Cheng, Y. , Moravec, C. S. , Penn, M. S. , Mazgalev, T. N. , & Thomas, J. D. (2005). Differences in left ventricular long‐axis function from mice to humans follow allometric scaling to ventricular size. The Journal of Physiology, 568(1), 255–265. 16002448 10.1113/jphysiol.2005.090779 PMC1474754

[eph13399-bib-0029] Potter, L. R. , Yoder, A. R. , Flora, D. R. , Antos, L. K. , & Dickey, D. M. (2009). Natriuretic peptides: Their structures, receptors, physiologic functions and therapeutic applications. Handbook Experimental Pharmacology, 191, 341–366. 10.1007/978-3-540-68964-5_15 PMC4855512 19089336

[eph13399-bib-0030] Qvigstad, E. , Moltzau, L. R. , Aronsen, J. M. , Nguyen, C. H. , Hougen, K. , Sjaastad, I. , Levy, F. O. , Skomedal, T. , & Osnes, J. B. (2010). Natriuretic peptides increase beta1‐adrenoceptor signalling in failing hearts through phosphodiesterase 3 inhibition. Cardiovascular Research, 85(4), 763–772. 19900965 10.1093/cvr/cvp364

[eph13399-bib-0031] Romano, F. D. , Naimi, T. S. , & Dobson, J. G., Jr. (1991). Adenosine attenuation of catecholamine‐enhanced contractility of rat heart in vivo. American Journal of Physiology, 260, H1635–H1639. 2035682 10.1152/ajpheart.1991.260.5.H1635

[eph13399-bib-0032] Sangaralingham, S. J. , Kuhn, M. , Cannone, V. , Chen, H. H. , & Burnett, J. C. (2023). Natriuretic peptide pathways in heart failure: Further therapeutic possibilities. Cardiovascular Research, 118(18), 3416–3433. 36004816 10.1093/cvr/cvac125 PMC9897690

[eph13399-bib-0033] Soeki, T. , Kishimoto, I. , Okumura, H. , Tokudome, T. , Horio, T. , Mori, K. , & Kangawa, K. (2005). C‐type natriuretic peptide, a novel antifibrotic and antihypertrophic agent, prevents cardiac remodeling after myocardial infarction. Journal of the American College of Cardiology, 45(4), 608–616. 15708711 10.1016/j.jacc.2004.10.067

[eph13399-bib-0034] Subramanian, H. , Froese, A. , Jonsson, P. , Schmidt, H. , Gorelik, J. , & Nikolaev, V. O. (2018). Distinct submembrane localisation compartmentalises cardiac NPR1 and NPR2 signalling to cGMP. Nature Communications, 9(1), 2446. 10.1038/s41467-018-04891-5 PMC6014982 29934640

[eph13399-bib-0035] Szaroszyk, M. , Kattih, B. , Martin‐Garrido, A. , Trogisch, F. A. , Dittrich, G. M. , Grund, A. , Abouissa, A. , Derlin, K. , Meier, M. , Holler, T. , Korf‐Klingebiel, M. , Volker, K. , Garfias Macedo, T. , Pablo Tortola, C. , Boschmann, M. , Huang, N. , Froese, N. , Zwadlo, C. , Malek Mohammadi, M. , …, & Heineke, J. (2022). Skeletal muscle derived Musclin protects the heart during pathological overload. Nature Communications, 13(1), 149. 10.1038/s41467-021-27634-5 PMC8748430 35013221

[eph13399-bib-0036] Wollert, K. C. , Yurukova, S. , Kilic, A. , Begrow, F. , Fiedler, B. , Gambaryan, S. , Walter, U. , Lohmann, S. M. , & Kuhn, M. (2003). Increased effects of C‐type natriuretic peptide on contractility and calcium regulation in murine hearts overexpressing cyclic GMP‐dependent protein kinase I. British Journal of Pharmacology, 140(7), 1227–1236. 14609817 10.1038/sj.bjp.0705567 PMC1574150

[eph13399-bib-0037] Zhao, Y. , Rafatian, N. , Feric, N. T. , Cox, B. J. , Aschar‐Sobbi, R. , Wang, E. Y. , Aggarwal, P. , Zhang, B. , Conant, G. , Ronaldson‐Bouchard, K. , Pahnke, A. , Protze, S. , Lee, J. H. , Davenport Huyer, L. , Jekic, D. , Wickeler, A. , Naguib, H. E. , Keller, G. M. , Vunjak‐Novakovic, G. , …, & Radisic, M. (2019). A platform for generation of chamber‐specific cardiac tissues and disease modeling. Cell, 176(4), 913–927.e18. 30686581 10.1016/j.cell.2018.11.042 PMC6456036

